# Unraveling Environmental Effects on Mitochondria

**Published:** 2010-07

**Authors:** Charles W. Schmidt

**Affiliations:** **Charles W. Schmidt**, MS, an award-winning science writer from Portland, ME, has written for *Discover Magazine*, *Science*, and *Nature Medicine*

Look into any cell today, and you’ll see remnants of ancient bacteria by the thousands. These mitochondria—tiny organelles in the cell that each possess their own DNA—have come under a growing scientific spotlight; scientists increasingly believe they play a central role in many, if not most, human illnesses. Exquisitely sensitive to environmental threats, mitochondria convert dietary sugars into a high-energy molecule—adenosine triphosphate (ATP)—that cells use as fuel. And when mitochondria falter, cells lose power, just as a flashlight dims when its batteries weaken. Now scientists are linking environmental interactions with the mitochondria to an array of metabolic and age-related maladies, including cancer, autism, type 2 diabetes, Alzheimer disease, Parkinson disease, and cardiovascular illness.

## Surge in Awareness

Mitochondrial disease was featured as a research theme during the Society of Toxicology’s (SOT) 2010 annual meeting. Michael Holsapple, who chaired SOT’s science program committee, attributes that decision to a growing realization among toxicologists that mitochondrial genomes are uniquely susceptible to oxidative damage and stress. “They’ve got a much higher percentage of coding DNA in contrast to nuclear genomes, which contain long noncoding regions,” explains Holsapple, who is executive director of the International Life Sciences Institute’s Health and Environmental Sciences Institute in Washington, DC. “And what we’re learning is that they play a significant role in neurodegenerative diseases and metabolic syndrome, which is a more amorphous designation that reflects an epidemic of obesity. We see this as cutting-edge science.”

Growing awareness of the role of mitochondria in health has sparked a surge in the number of scientists who study the topic. “A few years ago, there were just a handful of us,” says Kendall Wallace, a toxicologist and professor at the University of Minnesota Medical School. “Now we’ve got a critical mass of scientists with active research programs generating data.” But understanding the role of mitochondria in health and disease doesn’t come easily.

Each cell contains hundreds or even thousands of mitochondria. And cells falter only when their mitochondrial content (or “copy number”) drops below a critical threshold. Douglas Wallace, a professor at the University of California, Irvine, says mitochondrial research aims for a union between two fundamental components in biology: structural components (such as genes, proteins, tissues, and organs) and bioenergetic components (which relate to energy metabolism and how it sustains living systems). Mitochondria represent an interface between structure and energy, yet scientists have struggled with how to assess mitochondrial status, particularly *in vivo*.

These technical challenges complicate basic research and impede efforts to diagnose and treat mitochondrial problems. At an NIEHS-sponsored meeting held 25 June 2009, participants cited an urgent need for assays and biomarkers that detect early mitochondrial damage.^1^ Such tools might promote interventions during subclinical disease stages, the participants say. Bruce Cohen, a professor and neurologist at the Cleveland Clinic’s Neurological Institute and Taussig Cancer Center, says only a few hospitals have the expertise needed to diagnose mitochondrial conditions. Mitochondrial tests are expensive, he says, and they also lack what most doctors would consider to be adequate sensitivity (diagnosis of true cases) and specificity (accuracy in diagnosis).

Douglas Wallace adds that because clinicians don’t yet have tools for measuring energy flows in living organisms, many mitochondrial conditions go undiagnosed. “Research groups in the mitochondrial field are scrambling to get tools for measuring energy deficits, but they’re not available to doctors who see patients,” he says. “So from [the perspective of] the practice of medicine, if you don’t have the test, the problem seems to not exist. But it’s not true that what you don’t know won’t hurt you—these are real phenomena, and they cause real health problems.” Still, the field is making progress, and advances in mitochondrial research are generating insights into how the environment affects mitochondrial health and what we can do about it, Wallace says.

## An Expanding View of Mitochondrial Toxicology

Mitochondria can be thought of as processing centers on a path that begins with sunlight and ends with ATP. All the energy on Earth can be traced back to the sun and the way photosynthetic life forms absorb solar rays to power the production of glucose from water and carbon dioxide. The most fundamental fuel on the planet, glucose is a six-carbon sugar. Cells break it down further in the cytosol (their liquid interior) to produce smaller molecules, including, among others, a three-carbon acid called pyruvate and nicotinamide adenine dinucleotide (NADH).

During ATP production, pyruvate and NADH are drawn into the mitochondria, where they undergo a complex series of electrochemical reactions known collectively as oxidative phosphorylation. These aerobic reactions occur inside the mitochondria along what’s known as the electron transport chain (ETC). During oxidative phosphorylation, NADH transfers electrons to oxygen, and the energy released by that reaction is used to create ATP from a molecular precursor called adenosine diphosphate (ADP). Released into the cell, ATP is inherently unstable—enzymes break it apart to release energy for most biochemical reactions. And that regenerates ADP, which then returns to the mitochondria. According to NIEHS principal investigator William Copeland, humans typically create and consume their own weight in ATP every day.

Mitochondria also have their own genomes, relics of their ancient bacterial past. Sequenced by scientists in 1981, the human mitochondrial genome has more than 16,000 DNA base pairs (compared with 3 billion in the human nuclear genome) that code for 24 RNA molecules and 13 proteins.^2^ The nuclear genome also codes for roughly 1,500 mitochondrial proteins.

Beyond making ATP, mitochondria have other important functions. For instance, they generate reactive oxygen species (ROS), or free radicals, which participate in cell signaling and communication, particularly between nuclear and mitochondrial genes. Excessive ROS also can be toxic, and elevated levels can trigger a type of programmed cell death called apoptosis. Scientists have, in fact, linked asbestos-induced elevations in ROS to apoptosis in alveolar epithelial cells,^3^ which could explain how asbestos causes lung disease.

In yet another crucial function, mitochondria regulate intracellular stores of calcium, but sometimes that leads to too much of a good thing. When the mitochondria absorb excess calcium, they initiate apoptosis, and evidence suggests this activity in nerve cells might contribute to Alzheimer disease.^4^

Mitochondrial diseases generally have been thought of as a spectrum of primarily inherited conditions afflicting roughly 1 in every 2,000–5,000 people, says Cohen; about 1 in 200 people in the general population are thought to carry potentially pathogenic mitochondrial DNA mutations.^5^ Many mitochondrial conditions share common but nonspecific symptoms, such as fatigue and muscle pain. Others have more precise and debilitating features. For instance, MELAS (short for mitochondrial encephalomyopathy, lactic acidosis, and strokelike episodes), which is caused by inherited mutations in mitochondrial DNA, leads to seizures, migraines, and brain damage. Almost invariably fatal, the illness emerges when physical exertion overwhelms the body’s energy-generating capacity.

Beyond genetic causes, scientists now attach growing importance to environmental factors that trigger mitochondrial problems. In some cases, illness ensues only when chemical exposures interact with genetic risk factors. In one classic gene–environment interaction, people with an inherited mutation at allele 1555 of mitochondrial DNA (found in 1 of every 1,200 people) will have a high likelihood of deafness with exposure to aminoglycoside antibiotics such as gentamicin, says Cohen. Other genetic factors modify the severity of this effect. Cohen says a simple genetic test for the mutation is available and could be considered reasonable to add to newborn screening for metabolic disorders, so as to avoid aminoglyosides if at all possible in those carrying this mutation. Likewise, a number of mitochondrial DNA mutations predispose people to Leber hereditary optic neuropathy, a severe visual loss that usually occurs in young adults. Visual loss may be more frequent and severe in people who drink alcohol or smoke cigarettes; those who quit experience some return of visual function, Cohen adds. This condition also can be diagnosed with a genetic test.

## The Field Emerges

According to Dean Jones, a professor at Emory University School of Medicine, modern mitochondrial research dates back to the 1940s. That’s when scientists realized that when cyanide and carbon monoxide bind with hemoglobin in red blood cells, they take oxygen out of circulation, blocking energy production in the mitochondria. Over time, other aspects of mitochondrial function were revealed. Oxidative phosphorylation was described in more detail in the 1960s and calcium regulation in the 1970s, and by the 1990s, researchers had discovered that mitochondria coordinate apoptosis, which was a groundbreaking finding. Until then, it was assumed that cell death from mitochondrial dysfunction resulted mainly from failures in ATP production.

When scientists found that mitochondria release several proteins (including cytochrome C and apoptosis-inducing factor) to actively trigger cell death,^6^ the field was turned on its head. “That finding brought in a brand new audience of investigators,” Kendall Wallace says. “Suddenly, we had researchers involved in cell cycling, signaling, and cancer all working on the mitochondria; it really added to a growth of interest in the field.”

Another crucial development came when scientists detected organ toxicity in HIV/AIDS patients treated with azidothyramidine (AZT). This drug, commercialized during the late 1980s, transformed HIV/AIDS from a certain death sentence to a manageable chronic condition. But within a few years, as many as 30% of patients treated with AZT (and other drugs in its class) were developing complications such as weakening heart muscles and vision loss. Researchers soon implicated mitochondrial toxicity in these effects.^7^ AZT is designed to inhibit an enzyme, DNA polymerase, which is involved in HIV replication. But mitochondrial DNA polymerase gamma (pol γ) also is targeted, resulting in abnormal mitochondrial and cell function. As mitochondrial performance drops, cells revert back to glycolysis—a more primitive, less efficient mechanism—to generate ATP. That change in metabolism is reflected by a build-up of lactic acid, a by-product of glycolysis, which can be used as a biomarker of mitochondrial failure.^7^ In fact, recent evidence shows a link between autism and a build-up of lactic acid in serum, suggesting a possible role for mitochondrial dysfunction in the etiology of this disease.^8^

According to Yvonne Will, an associate research fellow at Pfizer Global Research and Development, the link between AZT and mitochondrial side effects raised the awareness that drugs could have mitochondrial liabilities. She says it was soon found that members of other drug families including the antilipidemic, antidiabetic, antidepressant, beta-blocker, and nonsteroidal anti-inflammatory drug classes also showed potential to induce mitochondrial side effects. This led to the development of high-throughput screens for mitochondrial damage that are now routinely deployed throughout the drug discovery process.

## Environmental Toxicology Takes Note

Compared with pharmaceutical research, environmental toxicology is only now getting up to speed on the effects of chemicals on mitochondrial functioning. Copeland says mitochondria are like canaries in a coalmine: susceptible to early-stage effects that predict cell and organ toxicity later. Research shows that mitochondrial DNA is uniquely susceptible to the damaging effects of ROS.^9^ These effects are more extensive and longer-lasting in mitochondrial DNA than they are in the nuclear genome, he adds, which suggests that relatively minor additional stress—alcohol, cigarette smoke, or other toxicants, for instance—could tip someone toward metabolic illness.

Joel Meyer, an assistant professor at Duke University, adds that, compared with its nuclear counterpart, mitochondrial DNA generally has less capacity to repair itself. It specifically can’t delete the large DNA helix–distorting adducts formed when mitochondrial DNA bases are damaged by mutagens such as polyaromatic hydrocarbons and ultraviolet radiation. The toxicologic implications of these adducts are now under investigation. “Our hypothesis is that [adducts lead] to altered transcription of mitochondrial genes, changes in mitochondrial copy number, mitochondrial mutations, and compromised ETC functioning,” Meyer says.

Another factor exacerbates the sensitivity of mitochondrial genetics, Copeland adds, that being reliance on just one enzyme—pol γ—for both replication and repair of mitochondrial DNA. In contrast, the nuclear genome has 15 such enzymes, he says. According to Copeland’s research, more than 85% of spontaneous mitochondrial DNA mutations can be traced back to errors in the *POLG* gene that encodes pol γ.^10^ Those mutations accumulate over time, so that with age individuals become less able to recover from environmental exposures.

This diminished capacity likely plays an important role in age-related illnesses, Copeland says. “It’s well known that mitochondrial DNA mutations and deletions accumulate during aging,” he explains. “Mouse models show that if you accelerate mitochondrial degeneration, you get premature aging. We already know that older people can’t run marathons as well as younger people and are more susceptible to environmental toxicants, possibly due to the accumulation of more damaged mitochondria.”

Copeland’s research has helped to identify approximately 200 *POLG* mutations in disease. Among them, more than 80 are linked to a neurologic and hepatic condition known as Alper disease, which typically afflicts young children, who rarely live past 10 years. A later-onset form of Alper disease can be triggered by environmental factors, Copeland says, particularly viruses. “But avoiding viruses is admittedly hard to do,” he adds.

Other environmental chemicals are thought to interact directly with the ETC, which has five distinct complexes (or transmembrane protein structures), each with its own task in energy production. Scientists Tim Greenamyre of the University of Pittsburgh and Todd Sherer of the Michael J. Fox Foundation for Parkinson’s Research have shown that several pesticides, including rotenone and pyridaben, alter complex I, and that the resulting oxidative stress damages cells.^11^ In fact, rotenone exposure is an experimental model for Parkinson disease in animals, which develop analogous symptoms following intravenous and subcutaneous administration of the chemical, as shown in unpublished research by Meyer. Several epidemiologic studies suggest an association between Parkinson disease and pesticide exposure,^12^ but scientists have yet to pinpoint a specific pesticide in disease onset.

The reality is that from an environmental perspective, these are still early days for mitochondrial toxicity research, says Meyer. In his opinion, although mitochondrial toxicants have been identified, the field still lacks a “slam-dunk” association between ambient chemical exposures and chronic toxicity and disease resulting from mitochondrial disruption, such as the one that exists for AZT and its side effects in HIV/AIDS patients. “That [discovery] was a defining event for the pharmaceutical industry, but we don’t have an equivalent for that in environmental health,” he says.

According to Kendall Wallace, exceptions could be the pesticides rotenone and cyanide, which inhibit ETC and deprive cells of ATP, and pentachlorophenol, which has been implicated in the weight loss associated with its use as an antifungal agent in wood treatments. “Broadening the definition of mitochondrial toxicity to include mitochondrial targets beyond the ETC brings many additional environmental agents into the fold,” he says. “Examples include fluoroacetate and fluoroacetamide, which inhibit the tricarboxylic acid cycle, and alkyl acids that inhibit the transport and/or oxidation of fats. Both of these metabolic pathways are localized within the matrix of the mitochondria, and both toxicities are manifested as a metabolic or bioenergetic disease.”

One thing that’s needed, Meyer says, are better ways to evaluate mitochondrial function in whole organisms. Chemical effects on isolated mitochondria can be detected using polymerase chain reaction methods that measure DNA damage, or with oxygen sensors that measure aerobic respiration. “But by comparison, measurements *in vivo* are more challenging because mitochondrial biology varies with tissues and developmental stage, Meyers says.^13^

## Energy Sense

According to Jones, growing acknowledgement of the role of mitochondria in disease makes intuitive sense. “Everything in our body is dependent on energy metabolism,” he says. “So if something alters that metabolism or the cell-death signaling pathways, you would expect this is going to have impacts on the full range of diseases. Just the fundamental act of being alive, our activity levels, behaviors, diet, and infection—everything factors in to this. The challenge for us is to identify the environmental risks that we can actually do something about.”

Until we gain a clearer understanding of which specific environmental agents damage mitochondria and how they do it, Cohen says it is probably safe to assume that eating a sensible, nutritious diet is one of the best protections against mitochondrial damage. “Overeating creates an increase in free radical formation and generates internal toxins,” he says. “My guess is that for the average person not living near a toxic waste dump there are more internal poisons from overeating than the person will be exposed to in everyday life. As my grandmother always said, eat your vegetables and get exercise.”

## For More Information

The United Mitochondrial Disease Foundation

http://www.umdf.org/

Mitochondrial DNA mutation database

http://www.mitomap.org/

DNA polymerase gamma gene mutation database

http://tools.niehs.nih.gov/polg/

## Mitochondrial Diseases

Alpers Disease

Barth Syndrome

Beta-Oxidation Defects

Carnitine Deficiency

Carnitine-Acyl-Carnitine Deficiency

Carnitine Palmitoyl Transferase (CPT) I Deficiency

Carnitine Palmitoyl Transferase (CPT) II Deficiency

Chronic Progressive External Ophthalmoplegia Syndrome

Coenzyme Q_10_ Deficiency

Complex I Deficiency

Complex II Deficiency

Complex III Deficiency

Complex IV Deficiency

Complex V Deficiency

Cytochrome C Oxidase (COX) Deficiency

Creatine Deficiency Syndromes

Kearns-Sayre Syndrome

Lactic Acidosis

Leber Hereditary Optic Neuropathy

Leigh Disease

Lethal Infantile Cardiomyopathy

Long-Chain 3-Hydroxyacyl-CoA Dehydrogenase Deficiency

Long-Chain Acyl-CoA Dehydrogenase Deficiency

Luft Disease

Medium-Chain Acyl-CoA Dehydrogenase Deficiency

Mitochondrial DNA Depletion

Mitochondrial Encephalopathy

Mitochondrial Encephalomyopathy, Lactic Acidosis, and Strokelike Episodes

Mitochondrial Myopathy

Mitochondrial Recessive Ataxia Syndrome

Multiple Acyl-CoA Dehydrogenase Deficiency/Glutaric Aciduria Type II

Myoclonic Epilepsy and Ragged-Red Fiber Disease

Myoneurogastrointestinal Disorder and Encephalopathy

Neuropathy, Ataxia, and Retinitis Pigmentosa

Pearson Syndrome

Pyruvate Carboxylase Deficiency

Pyruvate Dehydrogenase Deficiency

Short-Chain Acyl-CoA Dehydrogenase Deficiency

Short-Chain-3-hydroxyacyl-CoA Dehydrogenase Deficiency

Very Long-Chain Acyl-CoA Dehydrongenase Deficiency

Source: United Mitochondrial Disease Foundation

## Figures and Tables

**Figure:**
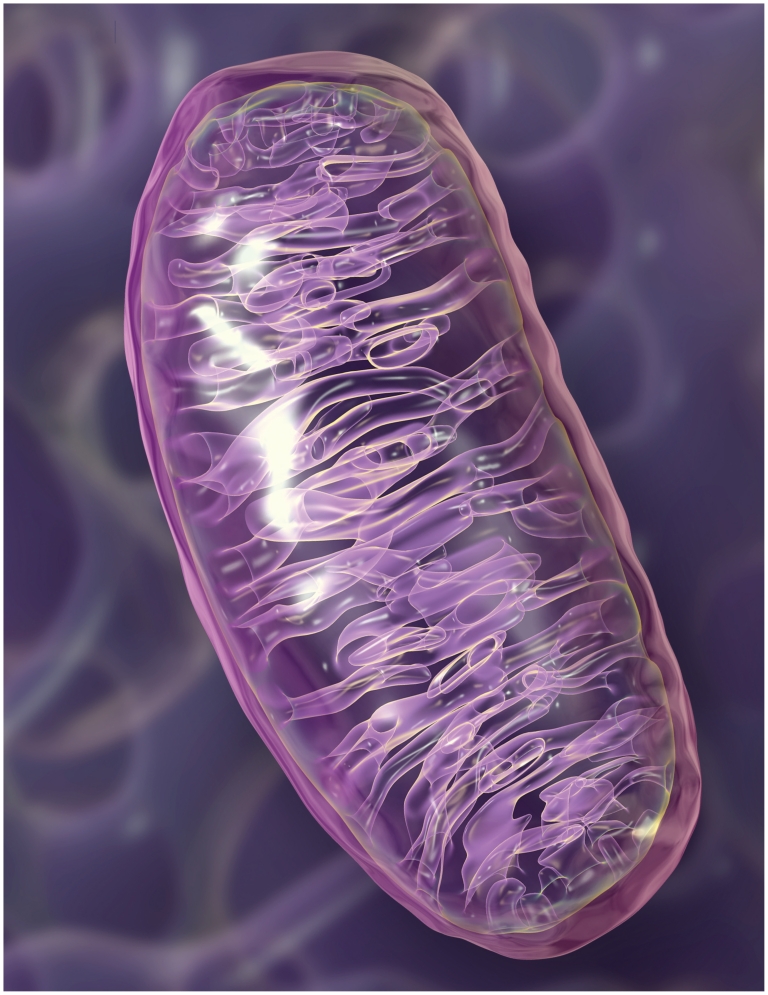
In 1962 Rolf Luft became the first clinician to recognize mitochondrial dysfunction as a cause of disease. Nearly 50 years later, the field of mitochondrial toxicology is getting a fresh boost with the realization that environmental agents may play a significant role in many mitochondrial diseases.

**Figure:**
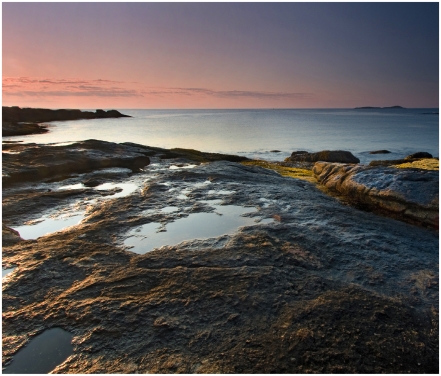
About two billion years ago, primitive seas swarmed with a simple life form on the verge of a change. Amounting to little more than some DNA wrapped in a membrane, this “proto-eukaryote” powered itself with a type of metabolism called glycolysis. Proto-eukaryotes were relics of an earlier time, when volcanic gases dominated the atmosphere. Now photosynthetic algae were on the scene, and they were filling the atmosphere with oxygen. The appearance of oxygen was transforming. Bacteria started burning it with a new, more efficient kind of metabolism—aerobic respiration—which gave them a competitive advantage. But not to be outdone, proto-eukaryotes simply engulfed the bacteria, to their mutual advantage: bacteria supplied their hosts with energy, and in exchange, the proto-eukaryote supplied nutrients and protection. And over the next 1.2 billion years, they co-evolved into eukaryotic cells—the basic units of plant and animal life.

**Figure:**
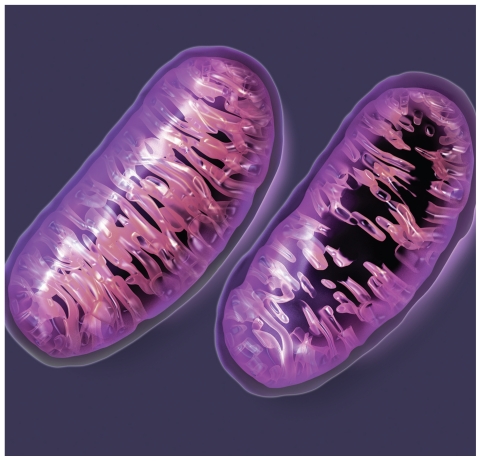
The distinctive folds of the inner membrane of each mitochondrion are known as cristae. The looping cristae offer maximum surface area for the important work of converting oxygen and sugars into energy via a series of reactions, with the electron transport chain producing the final energy payout. In a normal mitochondrion, the cristae fill the interior, but damaged or dysfunctional mitochondria lose cristae. If there is an error in any of the myriad steps involved in energy production, organ and system function can falter. Dozens of rare diseases have been shown to result from mitochondrial dysfunction (see list above). Several others—including Alzheimer disease, autism, cancer, cardiovascular disease, Parkinson disease, and type 2 diabetes—are suspected to involve the mitochondria.
